# Study on the knowledge, attitude and practice of single photon emission computed tomography among oncology healthcare professionals

**DOI:** 10.3389/fpubh.2024.1512686

**Published:** 2024-12-20

**Authors:** Min Yan, Xiang Cheng, Xinyu Li, Xiangting Jin, Ying Dai, Fanfan Li

**Affiliations:** ^1^Department of Oncology, The Second Affiliated Hospital of Anhui Medical University, Hefei, China; ^2^Anhui Medical University, Hefei, China; ^3^Department of Oncology Cancer Centre, Lu’an Hospital of Anhui Medical University, Lu’an, China; ^4^Department of Oncology, The First Affiliated Hospital of Anhui Medical University, Hefei, China

**Keywords:** single photon emission computed tomography, healthcare professional, knowledge, attitude, practice

## Abstract

**Background:**

Single photon emission computed tomography (SPECT) is becoming increasingly popular in oncology. This study endeavors to scrutinize the radiation protection knowledge, attitude, and practice (KAP) exhibited by healthcare professionals involved in this imaging modality.

**Methods:**

This cross-sectional study was conducted between September 23, 2023, and October 23, 2023, at the Second Affiliated Hospital of Anhui Medical University, the First Affiliated Hospital of Anhui Medical University, and the People’s Hospital of Liuan. Demographic data and KAP scores were acquired through the administration of questionnaires.

**Results:**

A total of 450 healthcare professionals participated in the study. Correlation analyses revealed significant positive correlations between knowledge and attitude, knowledge and practice, as well as attitude and practice. Multivariate analysis indicated that being over 40 years old was independently associated with good knowledge, as well as positive attitudes. Occupations as nurses and having no contact with SPECT patients were independently associated with a lower level of knowledge, as well as negative attitudes. Furthermore, being female, having an occupation as a nurse, and not having received relevant training were independently associated with negative practice.

**Conclusion:**

Oncology healthcare professionals had suboptimal knowledge, negative attitude and inactive practice towards radiation protection in SPECT.

## Introduction

SPECT (Single-Photon Emission Computed Tomography) is an advanced imaging technique that offers significant advantages in the early detection and evaluation of bone metastasis, particularly in lung cancer. By using a radioactive isotope tracer, such as 99mTc-MDP, SPECT enables a comprehensive assessment of bone metabolism, blood flow changes, and early-stage metastatic sites in a single imaging session. Its ability to integrate physiological, biochemical, functional, and metabolic scans makes it a valuable tool in identifying asymptomatic bone metastases, thus allowing for more precise treatment planning and improved patient outcomes (1–3). Bone metastasis is a common complication across various cancer types, notably breast and prostate cancers, and is associated with a higher risk of mortality. Early detection of bone metastasis in lung cancer is crucial for timely intervention. Although symptoms like bone pain, elevated blood alkaline phosphatase, or hypercalcemia can suggest bone involvement, conventional imaging methods, including CT scans, often lack the sensitivity needed to detect metastasis early (4–6). SPECT has an important clinical role in the evaluation of bone lesions in these patients (7). SPECT has also other important clinical applications, such as lung ventilation/perfusion studies (8), and sentinel node biopsy in breast cancer (9) and sentinel node biopsy in melanoma (10).

The Knowledge, Attitude, and Practices (KAP) survey serves as a diagnostic research tool, shedding light on a group’s understanding, beliefs, and actions concerning a specific subject, particularly within the domain of health literacy. This tool operates on the premise that knowledge positively influences attitudes, subsequently shaping behaviors (11–13). While existing research has primarily concentrated on the technical aspects of SPECT imaging and the formulation of radiation protection guidelines (14–16), there is a notable gap in the literature concerning the actual KAP levels among oncology healthcare professionals, especially in the context of SPECT. Understanding these factors is crucial for identifying areas where educational interventions may be necessary to enhance radiation safety. Given the radiation involved in SPECT, it is essential to understand the safety awareness and operational standards of the healthcare professionals using this technology to ensure the safety of both patients and medical staff. However, current research tends to emphasize the theoretical aspects of radiation protection, often overlooking the practical challenges healthcare professionals face in implementing these principles in their daily clinical practice.

This study focuses on assessing the KAP of radiation protection among oncology healthcare professionals, specifically in the context of SPECT. The aim is to understand their attitudes, knowledge of radiation safety, and acceptance of protective measures in SPECT. By enhancing the protection levels of healthcare professionals, ensuring radiation safety for SPECT patients, and minimizing risks for both patients and medical personnel, the study seeks to improve the overall quality of medical services.

## Materials and methods

### Study design and participants

This cross-sectional study was conducted between September 23, 2023, and October 23, 2023, at the Second Affiliated Hospital of Anhui Medical University, the First Affiliated Hospital of Anhui Medical University, and Lu’an Hospital of Anhui Medical University. Participants in the study comprised healthcare professionals specializing in oncology, including medical students. This study was approved by the Ethic Committee of the Second Affiliated Hospital of Anhui Medical University (YX2023-153), and all participants provided written informed consent.

Inclusion criteria were defined as follows: (1) Engagement in work related to oncology; (2) Regular involvement with SPECT patients in daily professional activities; (3) A certain level of understanding of SPECT; and (4) Ability to provide genuine responses to the relevant questionnaire. Exclusion criteria encompassed: (1) Non-oncology-related personnel; (2) Lack of regular involvement with SPECT patients in daily work; and (3) Inability to provide authentic information.

The distribution of questionnaires to research subjects occurred through WeChat and QQ groups. The study encompassed a total of seven tertiary comprehensive hospitals, with specific involvement from three of these hospitals. The First Affiliated Hospital of Anhui Medical University comprised 4 wards in the oncology department and 4 wards in the radiotherapy department. The Second Affiliated Hospital of Anhui Medical University included 5 wards in the oncology department. People’s Hospital of Liuan comprised 2 wards in the oncology department and 1 ward in the radiotherapy department. The total number of healthcare professionals, including medical students, was approximately 700.

### Sample size determination

The sample size was calculated using the following formula:


$$n=\frac{z^{2}p\left(1-p\right)}{d^{2}}$$

where z = 1.96 at 5% level of significance and 5%acceptable margin of error (d = 0.05). The proportion of the expected population based on previous studies or pilot studies is set at 50%. Based on the above, the sample size was calculated as 384 (17).

According to the inclusion and exclusion criteria, a total of 654 eligible medical staff from participating hospitals were identified. Ultimately, 608 questionnaires were distributed, and 450 valid responses were received, yielding an effective response rate of 74% (actual data to be filled). All data were collected in accordance with principles of confidentiality and voluntary participation, strictly for research purposes. For sample size calculation, prior studies in similar fields were referenced, with a significance level of *α* = 0.05 and a statistical power of 80%. Based on the objectives of the KAP survey in this study, the minimum required sample size was 384. The actual effective sample size of 450 met this requirement, ensuring sufficient statistical power for the analysis.

### Procedures

Following the design of the questionnaire, feedback from three oncology experts was sought and incorporated for refinements. Subsequently, a pilot study was conducted with a limited sample size (30 responses), yielding a reliability coefficient of 0.814 and a Kaiser-Meyer-Olkin (KMO) measure of 0.815. Details of the questionnaire can be found in the Supplementary document “Questionnaire”.

The final questionnaire, presented in Chinese, encompasses data collection across four dimensions. It comprises 9 questions for basic information, 15 items for the knowledge dimension, 9 items for the attitude dimension, and 7 items for the practice dimension. During statistical analysis, scores were assigned based on the number of response options for each item. For instance, in the knowledge dimension, a correct answer was allocated 1 point, while an incorrect or unclear response received 0 points. In the attitude and practice dimensions, scores were assigned in descending order (positive to negative), with the final total score falling within a specified range (from lowest to highest). Items that could not be scored were treated as distinct categorical variables. Achieving scores exceeding 70% of the maximum in each section denoted sufficient knowledge, positive attitude, and proactive practice (18).

### Statistical analysis

The sample size determination relied on a prior study (19), and subsequent descriptive analysis encompassed demographic information and dimension scores. Initial normality tests guided the choice between mean and standard deviation or median, 25th percentile, and 75th percentile representation for dimension scores. Count data for demographics and question responses were expressed as *N*(%). Dimension score differences among subjects with varied demographics were assessed using the Wilcoxon-Mann–Whitney test for non-normally distributed two-group comparisons, and Kruskal-Wallis analysis for three or more groups. Pearson correlation coefficients were used for normal distribution in correlation analyses; otherwise, Spearman coefficients were applied. In this analysis, we used the median score as the cut-off value for classification in both univariate and multivariate logistic regression analyses. Further details on how the median was determined and applied are provided in the Methods section to clarify the rationale and ensure consistency in the statistical approach. Variables inclusion in multivariate regression relied on univariate significance (*p* < 0.1), rounding *p* values to three decimal places, and considering *p* < 0.05 as statistically significant. A total of 608 questionnaires were collected; 14 were excluded for completion times, 80 for repeated IP addresses, and 64 for incomplete responses, resulting in 450 valid questionnaires.

## Results

### Demographic characteristics

**Figure 1:**
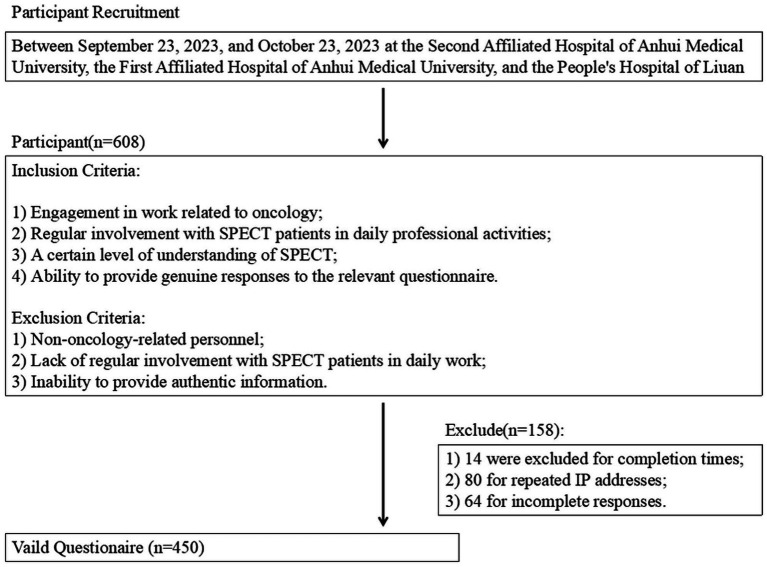
Flow chart.

Among the healthcare professionals who participated in the study, 608 questionnaires were collected. Of these, 14 questionnaires were excluded because they were completed in less than 60 s or more than 1800 s, 80 questionnaires were excluded due to repeated IP addresses, and 64 questionnaires were incomplete, resulting in a total of 450 valid questionnaires (Figure 1). Of the valid respondents, 310 (68.9%) were female, 259 (57.6%) were aged 30 years and below, 279 (62.0%) were doctors, and 303 (67.3%) had worked in oncology for 1–5 years. In addition, 321 (71.3%) had contact with patients who required SPECT, while 339 (75.3%) had no SPECT-related training. The median (25th percentile, 75th percentile) score of knowledge, attitude, and practice were 10 (9, 20), 27 (21, 22) and 18 (12, 18) separately. Analyses of differences in demographic characteristics showed that differences in gender, occupation, education, receipt of relevant training, and contact with SPECT patients were more likely to have differences in knowledge, attitude, and practice scores. In addition, healthcare professionals with different age, professional title, years of work experience in oncology were more likely to have different levels of knowledge (*p* < 0.005) (Table 1). There were 54.1, 52.7, and 51.7%, respectively, had knowledge, attitude, and practice scores ≥ the median (Table 2).

**Table 1:** Baseline sheet.

	N (%)	Knowledge (K)		Attitude (A)		Practice (P)	
		Median (25% quartile, 75% quartile)	*P*	Median (25% quartile, 75% quartile)	*P*	Median (25% quartile, 75% quartile)	*P*
**Total**	450	10 (8, 12)		27 (25, 29)		18 (15, 21)	
**Gender**			0.038		0.006		<0.001
Male	140 (31.1)	11 (8, 13)		27 (26, 29)		20 (17, 22)	
Female	310 (68.9)	10 (8, 12)		26 (24, 28)		18 (14, 21)	
**Age**			<0.001		0.605		0.900
30 years old and below	259 (57.6)	10 (7, 12)		26 (25, 28)		18 (15, 21)	
31–40 years old	169 (37.6)	11 (8, 12)		27 (25, 29)		19 (15, 22)	
Above 40 years old	22 (4.9)	12.5 (11, 14)		27 (26, 28)		18 (16, 20)	
**Occupation**			<0.001		<0.001		<0.001
Doctor	279 (62.0)	11 (9, 13)		27 (25, 29)		19 (16, 22)	
Nurse	109 (24.2)	9 (7, 11)		25 (23, 28)		16 (12, 20)	
Other	62 (13.8)	10 (7, 11)		27 (25, 28)		18 (13, 22)	
**Education**			<0.001		<0.001		0.008
Undergraduate and below	233 (51.8)	9 (7, 12)		26 (24, 28)		17 (14, 21)	
Postgraduate and above	217 (48.2)	11 (9, 13)		27 (25, 29)		19 (16, 21)	
**Professional title**			<0.001		0.117		0.324
Practising doctor/nurse and below	219 (48.7)	10 (7, 12)		27 (25, 29)		19 (15, 21)	
Attending doctor/Head nurse	132 (29.3)	10.5 (8, 12)		26 (24, 29)		18 (14.5, 21.5)	
Chief or Associate chief doctor/nurse	40 (8.9)	12.5 (11, 14)		27 (26, 29)		19 (17, 22)	
Other	59 (13.1)	10 (7, 11)		26 (24, 28)		18 (15, 20)	
**Years of work experience in oncology**			0.003		0.930		0.343
1–5 years	303 (67.3)	10 (8, 12)		27 (25, 28)		18 (15, 21)	
5–10 years	67 (14.9)	11 (9, 13)		26 (25, 30)		19 (16, 21)	
More than 10 years	80 (17.8)	11 (8, 14)		27 (24, 28)		18 (14, 21.5)	
**Nature of medical institution you work in**			0.459		0.545		0.119
Tertiary A	390 (86.7)	10 (8, 12)		27 (25, 28)		18 (15, 21)	
Other	60 (13.3)	11 (8, 12)		27 (25, 30)		19 (16, 22)	
**Received relevant training**			<0.001		0.009		<0.001
Yes	111 (24.7)	12 (9, 14)		27 (25, 30)		22 (19, 23)	
No	339 (75.3)	10 (7, 12)		26 (25, 28)		18 (14, 20)	
**Contact with SPECT patients**			<0.001		<0.001		0.002
Yes	321 (71.3)	11 (9, 13)		27 (25, 29)		19 (15, 22)	
No	129 (28.7)	8 (6, 10)		26 (23, 28)		18 (14, 20)	

**Table 2:** Score situation.

	Median	25% quintile	75% quintile	Minimum value	Maximum value	< Median *N* (%)	≥ Median *N* (%)
Knowledge dimension	10	8	12	0	16	193 (42.9)	257 (57.1)
Attitude dimension	27	25	29	13	34	215 (47.8)	235 (52.2)
Practice dimension	18	15	21	7	24	190 (42.2)	260 (57.8)

### Knowledge, attitudes, and practices

In the knowledge dimension, the highest proportion of participants answered correctly (77.3%) to the question “As SPECT patients carry radioactive substances, should they avoid close contact with pregnant women and infants?” (K9). On the contrary, for the question “To reduce radiation exposure for both patients and healthcare professionals, what is the most appropriate scanning time range? “(K6), only 2.9% of healthcare professionals answered correctly (Table 3A).

**Table 3:** Distribution of scores across dimensions.

(A) Knowledge dimension	
	Right option, *N* ()%
Do you have a basic understanding of the fundamental principles of Single Photon Emission Computed Tomography (SPECT)?	69 (15.3)
Are you aware of the advantages and limitations of SPECT?	203 (45.1)
In the diagnosis of tumors, under what circumstances do you believe SPECT should not be performed?	
Assessment of bone metastases Bone pain assessment	122 (27.1) 221 (49.1)
Bone tumor assessment Evaluation of treatment effect Asymptomatic screening	111 (24.7) 168 (37.3) 301 (66.9)
Does tumor tissue absorb more tracer?	336 (74.7)
Which cancers are more prone to causing bone metastasis?	
Breast cancer	364 (80.9)
Lung cancer	385 (85.6)
Liver cancer	202 (44.9)
Pancreatic cancer	124 (27.6)
Bowel cancer	108 (24.0)
To reduce radiation exposure for both patients and healthcare professionals, what is the most appropriate scanning time range?	13 (2.9)
Is it necessary to undergo regular SPECT scans for both early and late-stage breast cancer?	207 (46.0)
Does SPECT imaging, which requires the injection of radioactive isotopes, pose a significant radiation hazard to patients?	237 (52.7)
As SPECT patients carry radioactive substances, should they avoid close contact with pregnant women and infants?	348 (77.3)
After completing a SPECT examination, how much social distance should patients maintain from others?	121 (26.9)
Is isolation required after completing a SPECT examination?	253 (56.2)
Should patients drink more water and urinate frequently after completing a SPECT examination?	337 (74.9)
Approximately how fast is the metabolism of radioactive isotopes in the body of a SPECT patient?	128 (28.4)
Does impaired kidney function affect SPECT results?	233 (51.8)
Can patients who have taken bismuth-containing medications (such as Pepto-Bismol) or undergone X-ray examinations with barium contrast agents in the past four weeks undergo SPECT examinations?	215 (47.8)

(B) Attitude dimension					
	A	B	C	D	E
How do you perceive the application value of SPECT in the diagnosis and treatment of tumors?	Very valuable 189 (42.0)	Valuable 223 (49.6)	General 35 (7.8)	Worthless 1 (0.2)	Completely worthless 2 (0.4)
Are you willing to recommend or participate in the use of SPECT technology?	Very willingly 116 (25.8)	Willing 192 (42.7)	Neutrality 130 (28.9)	Unwillingness 11 (2.4)	Very reluctant 1 (0.2)
Do you believe that SPECT is helpful in improving the diagnostic efficiency and treatment outcomes of cancer patients?	Very helpful 166 (36.9)	It helps 259 (57.6)	Less helpful 18 (4.0)	Talk better than nothing 5 (1.1)	Not at all helpful 2 (0.4)
Do you think the radiation risk posed by SPECT to patients is a cause for concern?	Yes: 396 (88.0)	No: 54 (12.0)	/	/	/
After a SPECT examination, are you concerned that the radiation carried by the patient may impact your health?	Not at all worried 27 (6.0)	Do not worry 91 (20.2)	Neutrality 155 (34.4)	Worry 154 (34.2)	Very worried 23 (5.1)
Do you think it is possible to reduce the frequency of SPECT examinations?	Totally disagree 20 (4.4)	Disagree 49 (10.9)	Neutrality 217 (48.2)	Consent 154 (34.2)	Strongly agree 10 (2.2)
Do you believe that other examination methods such as CT or magnetic resonance imaging (MRI) are safer and can replace SPECT examinations?	Totally disagree 33 (7.3)	Disagree 204 (45.3)	Neutrality 172 (38.2)	Consent 40 (8.9)	Strongly agree 1 (0.2)
For routine SPECT procedures, do you feel that radiation protection measures are effective enough?	Yes: 275 (61.1)	No: 175 (38.9)	/	/	/
Do you think that in future clinical practice, SPECT technology will play a more significant role?	Yes: 365 (81.1)	No: 85 (18.9)	/	/	/

(C) Practice dimension					
	Yes	No			
Have you used SPECT technology in clinical practice?	237 (52.7)	213 (47.3)	/	/	/
	Definitely	Usually	Occasionally	Not at all	
When communicating with patients, do you explain the radiation safety and protection measures associated with SPECT?	154 (34.2)	166 (36.9)	76 (16.9)	54 (12.0)	/
	Definitely	Often	Usually	Occasionally	Not at all
Do you combine explanations of SPECT with other imaging examinations?	151 (33.6)	157 (34.9)	77 (17.1)	29 (6.4)	36 (8.0)
	Yes	No			
During the SPECT operation or after being informed that a patient has undergone SPECT, do you take appropriate protective measures?	296 (65.8)	154 (34.2)	/	/	/
Before conducting SPECT, do you follow strict preparation procedures and operational protocols?	291 (64.7)	159 (35.3)	/	/	/
Has the result of SPECT had a significant impact on your clinical diagnostic decisions?	364 (80.9)	86 (19.1)	/	/	/
Have you received training on accidents and emergency procedures related to SPECT?	152 (33.8)	298 (66.2)	/	/	/

When it comes to attitudes related to SPECT, 88.0% of healthcare professionals believe that the risk of radiation to patients from SPECT should be a concern (A4). Concurrently, 57.6% reported that they considered SPECT to be more useful in improving the diagnostic efficiency and therapeutic outcomes of oncology patients (A3), and 42.7% were willing to recommend or participate in the usage of this technology (A2). Regarding the impact of patient-carried radiation on the participants’ own health (A5) and the possibility of reducing the times of SPECT (A6), 34.4 and 48.2% were neutral, respectively. In addition, 61.1% believed that current radiation protection measures were adequate (A8), and 81.1% believed that SPECT would play a more important role in clinical practice in the future (A9) (Table 3B).

The answer to the practice question revealed that 52.7% of healthcare professionals have used SPECT in their clinical practice (P1), 65.8% protect themselves when potentially exposed (P4), and 64.7% follow rigorous preparations and procedures before performing SPECT (P5). Further, 80.9% indicated that SPECT results had a significant impact on their clinical diagnostic decisions (P6). It is important to note that 66.2% reported that they had never received training on SPECT-related accidents and emergency handling (P7), indicating a significant risk (Table 3C).

### Correlation analysis and multivariate logistic regression

Correlation analyses shown that significant positive correlations were found between knowledge and attitude (*r* = 0.333, *p* < 0.001), knowledge and practice (*r* = 0.333, *p* < 0.001), as well as attitude and practice (*r* = 0.430, *p* < 0.001), respectively (Table 4).

**Table 4:** Correlation analysis.

	Knowledge	Attitude	Practice
Knowledge	1.000	0.333 (*P* < 0.001)	0.330 (*p* < 0.001)
Attitude	0.333 (*P* < 0.001)	1.000	0.430 (*P* < 0.001)
Practice	0.330 (*P* < 0.001)	0.430 (*P* < 0.001)	1.000

Variables with *p* < 0.1 in the univariate analysis were included in the multivariate analysis, which showed that being over 40 years was independently associated with good knowledge (OR = 5.647, 95% CI: [1.623–19.650], *p* = 0.007) as well as positive attitudes (OR = 2.751, 95% CI: [1.012–7.482], *p* = 0.047). An occupation as a nurse (OR = 0.308, 95% CI: [0.185–0.514], *p* < 0.001 and OR = 0.314, 95% CI: [0.192–0.514], *p* < 0.001) and no contact with SPECT patients (OR = 0.226, 95% CI: [0.140–0.364], *p* < 0.001 and OR = 0.440, 95% CI: [0.282–0.684], *p* < 0.001) were independently associated with a lower level of knowledge as well as negative attitudes (OR < 1, *p* < 0.005) (Tables 5A,B). Further, being female (OR = 0.627, 95% CI: [0.393–0.999], *p* = 0.049), an occupation as a nurse (OR = 0.362, 95% CI: [0.219–0.597], *p* < 0.001), and not having received relevant training (OR = 0.284, 95% CI: [0.169–0.478], *p* < 0.001) were independently associated with negative practice (Table 5C).

**Table 5:** Univariate and multivariate regression analysis.

(A) Knowledge dimension					
Cut-off value: ≥10/<10		Univariate		Multivariate (*P* < 0.1)	
	No.	OR (95%CI)	*P*	OR (95%CI)	*P*
Gender					
Male	86/140	Ref.			
Female	171/310	0.772 (0.514, 1.161)	0.214		
Age					
30 years old and below	133/259	Ref.		Ref.	
31–40 years old	106/169	1.594 (1.073, 2.368)	0.021	1.538 (0.971, 2.434)	0.066
Above 40 years old	18/22	4.263 (1.404, 12.942)	0.010	5.647 (1.623, 19.650)	0.007
Occupation					
Doctor	183/279	Ref.		Ref.	
Nurse	42/109	0.329 (0.208, 0.520)	<0.001	0.308 (0.185, 0.514)	<0.001
Other	32/62	0.560 (0.321, 0.976)	0.041	0.714 (0.388, 1.313)	0.279
Education					
Undergraduate and below	108/233	Ref.			
Postgraduate and above	149/217	2.536 (1.725, 3.729)	<0.001		
Professional title					
Practising doctor/nurse and below	114/219	Ref.			
Attending doctor/head nurse	80/132	1.417 (0.914, 2.197)	0.119		
Chief or associate chief doctor/nurse	33/40	4.342 (1.842, 10.236)	0.001		
Other	30/59	0.953 (0.536, 1.694)	0.869		
Years of work experience in oncology					
1–5 years	161/303	Ref.			
5–10 years	49/67	2.401 (1.337, 4.312)	0.003		
More than 10 years	47/80	1.256 (0.763, 2.069)	0.370		
Nature of medical institution you work in					
Tertiary A	219/390	Ref.			
Other	38/60	1.349 (0.769, 2.366)	0.297		
Received relevant training					
Yes	82/111	Ref.		Ref.	
No	175/339	0.377 (0.235, 0.606)	<0.001	0.600 (0.355, 1.014)	0.056
Contact with SPECT patients					
Yes	220/321	Ref.		Ref.	
No	37/129	0.185 (0.118, 0.289)	<0.001	0.226 (0.140, 0.364)	<0.001

(B) Attitude dimension					
Cut-off value: ≥27 /<27		Univariate		Multivariate (*P* < 0.1)	
	No.	OR (95%CI)	*P*	OR (95%CI)	*P*
Gender					
Male	87/140	Ref.			
Female	148/310	0.557 (0.370, 0.837)	0.005		
Age					
30 years old and below	128/259	Ref.		Ref.	
31–40 years old	92/169	1.223 (0.829, 1.803)	0.310	1.393 (0.909, 2.135)	0.128
Above 40 years old	15/22	2.193 (0.866, 5.556)	0.098	2.751 (1.012, 7.482)	0.047
Occupation					
Doctor	163/279	Ref.		Ref.	
Nurse	34/109	0.323 (0.202, 0.516)	<0.001	0.314 (0.192, 0.514)	<0.001
Other	38/62	1.127 (0.641, 1.980)	0.678	1.388 (0.773, 2.493)	0.272
Education					
Undergraduate and below	104/233	Ref.			
Postgraduate and above	131/217	1.889 (1.298, 2.749)	0.001		
Professional title					
Practising doctor/nurse and below	114/219	Ref.			
Attending doctor/head nurse	64/132	0.867 (0.563, 1.336)	0.517		
Chief or Associate chief doctor/nurse	28/40	2.149 (1.039, 4.443)	0.039		
Other	29/59	0.890 (0.501, 1.583)	0.692		
Years of work experience in oncology					
1–5 years	161/303	Ref.			
5–10 years	31/67	0.759 (0.447, 1.291)	0.310		
More than 10 years	43/80	1.025 (0.625, 1.680)	0.922		
Nature of medical institution you work in					
Tertiary A	201/390	Ref.			
Other	34/60	1.230 (0.711, 2.127)	0.460		
Received relevant training					
Yes	67/111	Ref.			
No	168/339	0.645 (0.417, 0.998)	0.049		
Contact with SPECT patients					
Yes	188/321	Ref.		Ref.	
No	47/129	0.405 (0.266, 0.618)	<0.001	0.440 (0.282, 0.684)	<0.001

(C) Practice dimension					
Cut-off value: ≥18 /<18		Univariate		Multivariate (*P* < 0.1)	
	No.	OR (95%CI)	*P*	OR (95%CI)	*P*
Gender					
Male	99/140	Ref.		Ref.	
Female	161/310	0.447 (0.292, 0.686)	<0.001	0.627 (0.393, 0.999)	0.049
Age					
30 years old and below	146/259	Ref.			
31–40 years old	101/169	1.150 (0.776, 1.704)	0.487		
Above 40 years old	13/22	1.118 (0.462, 2.708)	0.805		
Occupation					
Doctor	184/279	Ref.		Ref.	
Nurse	40/109	0.299 (0.189, 0.475)	<0.001	0.362 (0.219, 0.597)	<0.001
Other	36/62	0.715 (0.408, 1.254)	0.242	0.791 (0.438, 1.428)	0.436
Education					
Undergraduate and below	116/233	Ref.			
Postgraduate and above	144/217	1.990 (1.359, 2.913)	<0.001		
Professional title					
Practising doctor/nurse and below	128/219	Ref.			
Attending doctor/head nurse	72/132	0.853 (0.552, 1.319)	0.475		
Chief or Associate chief doctor/nurse	29/40	1.874 (0.890, 3.945)	0.098		
Other	31/59	0.787 (0.442, 1.402)	0.416		
Years of work experience in oncology					
1–5 years	176/303	Ref.			
5–10 years	42/67	1.212 (0.703, 2.091)	0.489		
More than 10 years	42/80	0.798 (0.486, 1.308)	0.370		
Nature of medical institution you work in					
Tertiary A	220/390	Ref.			
Other	40/60	1.545 (0.871, 2.741)	0.136		
Received relevant training					
Yes	88/111	Ref.		Ref.	
No	172/339	0.269 (0.162, 0.446)	<0.001	0.284 (0.169, 0.478)	<0.001
Contact with SPECT patients					
Yes	195/321	Ref.			
No	65/129	0.656 (0.435, 0.990)	0.045		

## Discussion

The findings of this study indicate that oncology healthcare professionals exhibit suboptimal knowledge, negative attitudes, and inactive practices regarding radiation protection in SPECT. These results not only have implications for the operational behavior of healthcare workers but also significantly impact patient safety, the quality of care, and long-term health outcomes. To address these issues, the study recommends targeted educational interventions aimed at enhancing radiation protection practices among oncology healthcare professionals involved in SPECT. Key focus areas include healthcare professionals under 40, nurses, those without direct patient contact, and females. Tailored training programs addressing specific knowledge gaps and emphasizing protocol adherence are recommended to improve overall practice.

The study reveals deficiencies in medical management, primarily due to a lack of comprehensive SPECT-related training and experience disparities among healthcare professionals. This shortfall contributes to lower knowledge, attitude, and practice scores, indicating a broader systemic issue in ongoing professional development. Furthermore, the study reveals demographic disparities, with female healthcare workers and those with less direct contact with SPECT patients displaying lower proficiency. To address these issues, it’s crucial to implement mandatory, comprehensive training programs encompassing both theoretical and practical aspects of SPECT, tailored to different roles within the healthcare team. Additionally, fostering a culture of continuous learning through regular workshops and peer-led training sessions can help mitigate the experience gap, particularly for those less exposed to SPECT.

The median scores for knowledge, attitude, and practice were 10 (9, 20), 27 (21, 22), and 18 (12, 18), respectively, indicating room for improvement in all dimensions. A notable finding is the significant positive correlations between knowledge, attitude, and practice, reinforcing the interconnected nature of these components. Comparisons across demographic characteristics highlight variations in knowledge, attitude, and practice scores, emphasizing the need for targeted interventions. For instance, older age was independently associated with good knowledge and positive attitudes, while being a nurse and lacking contact with SPECT patients correlated with lower knowledge and negative attitudes. These results align with existing literature, emphasizing the influence of demographic factors on radiation protection awareness and practices among healthcare professionals (23).

In the knowledge dimension, this study identified significant gaps among oncology healthcare professionals regarding the fundamental principles and safety measures associated with SPECT technology. The lack of understanding, particularly in areas such as radiation metabolism, optimal scanning times, and patient management, indicates a need for more focused educational initiatives. Recent studies have highlighted strategies to address these gaps in radiation safety knowledge. For instance, a study propose comprehensive measures for advancing radiation protection and safety systems in nuclear medicine, which could be adapted to address these deficiencies in SPECT technology training (24). To address these deficiencies, it is recommended to implement targeted training programs that emphasize practical knowledge application. For instance, integrating case-based learning modules that simulate real-world scenarios involving SPECT can enhance the comprehension and retention of key concepts. Additionally, creating specialized workshops that focus on the nuances of radiation safety in SPECT, particularly tailored to different professional roles (e.g., technicians vs. physicians), can ensure that training is relevant and immediately applicable (21, 25). Reference to prior studies has shown that interactive training sessions significantly improve knowledge retention in similar contexts (26, 27).

In terms of attitude, although most healthcare professionals acknowledge the importance of SPECT in oncology, there is a notable lack of confidence in current radiation safety measures and a considerable concern about the potential risks posed by patients who have undergone SPECT. To improve these attitudes, it is crucial to implement regular feedback loops where healthcare professionals can share concerns and experiences related to radiation safety, which can then be addressed in subsequent training or guideline updates. Establishing a radiation safety mentorship program, where less experienced staff are paired with experts, can also help alleviate concerns and build confidence in safety protocols. Furthermore, promoting a culture of safety through regular, data-driven updates on the efficacy of existing safety measures, supported by transparent incident reporting and resolution processes, can reinforce the perceived value and effectiveness of radiation protection practices (22, 28).

In the practice dimension, while some healthcare professionals adhere to basic radiation protection guidelines during SPECT procedures, there is a significant portion that does not fully comply with established protocols, particularly regarding emergency procedures. To enhance compliance, it is recommended to incorporate mandatory, hands-on training sessions that simulate emergency scenarios involving radiation exposure. These sessions should be followed by debriefings that allow participants to reflect on their actions and learn from any mistakes in a controlled environment (29–31). Additionally, implementing periodic audits of radiation safety practices, with constructive feedback and targeted corrective actions, can help maintain high standards of safety. Specific strategies might include the use of real-time monitoring tools that alert staff when safety protocols are not followed, supported by immediate corrective training (32, 33). Furthermore, tailoring these interventions to the specific needs of different professional groups—such as differentiating training for those who primarily perform SPECT versus those who occasionally encounter it—can ensure that all healthcare professionals receive the most relevant and effective guidance (34).

Multivariate analysis further illuminates factors independently associated with knowledge, attitudes, and practices. Age over 40 emerged as a positive predictor of good knowledge and positive attitudes, aligning with studies emphasizing the cumulative experience and continuous learning associated with age (35, 36). Conversely, being a nurse and lacking contact with SPECT patients were linked to lower knowledge and negative attitudes, emphasizing the need for targeted interventions in these subgroups. Additionally, being female, working as a nurse, and lacking relevant training were independently associated with negative practices, emphasizing the role of gender and education in shaping healthcare professionals’ adherence to radiation protection protocols. These findings underscore the need for tailored educational programs targeting specific demographic groups and professional categories.

One limitation of this study is its cross-sectional design, which allows for the identification of associations but not causation. Additionally, the research was conducted in specific healthcare settings, potentially limiting the generalizability of the findings to other contexts. The reliance on self-reported data through questionnaires introduces the possibility of response bias, and the study’s focus on a specific geographical area may affect the external validity of the results. Furthermore, the study’s scope did not explore the impact of continuous professional development or specific training programs, and the reliance on healthcare professionals’ self-reported practices might not fully reflect their actual behavior in clinical settings. Moreover, the study did not examine whether there is a knowledge gap between youth who participate in similar sports without experiencing ACL injuries and those who have experienced ACL injuries, a potentially significant area for future investigation. Additionally, similar studies, such as the QUADRANT study (37), have provided systematic approaches for detecting knowledge, attitude, and practice deficiencies in medical fields, which could inform the design of future research. The use of online surveys and convenience sampling, while practical, may also introduce biases due to limited access for certain demographics and the accuracy of self-reported responses.

In conclusion, oncology healthcare professionals had suboptimal knowledge, negative attitude and inactive practice towards radiation protection in SPECT. Improving radiation protection practices among oncology healthcare professionals involved in SPECT can be achieved through targeted educational interventions. Specifically, efforts should focus on healthcare professionals under 40, nurses, and those without direct SPECT patient contact. Tailored training programs addressing these groups’ knowledge gaps and attitudes are recommended, emphasizing increased awareness and adherence to radiation protection protocols.

## Data Availability

The original contributions presented in the study are included in the article/Supplementary material, further inquiries can be directed to the corresponding authors.
