# Supplementary material for: Study on the knowledge, attitude and practice of single photon emission computed tomography among oncology healthcare professionals
Source: Front Public Health. 2024 Dec 20;12:1512686. doi: 10.3389/fpubh.2024.1512686 (PMC11695331; doi:10.3389/fpubh.2024.1512686)
Supplement: Supplementary file 1 [file Table_1.DOCX]

Dear Healthcare Professional,

Hello!

We are researchers from the Second Affiliated Hospital of Anhui Medical University, and we sincerely invite you to participate in our research project! This study aims to gather information to enhance our understanding of bone scans (SPECT), which will serve as a basis for formulating scientific intervention strategies in the future.

To participate, you only need to complete this questionnaire based on your actual situation. The contents of the questionnaire will be kept confidential, and your personal information will not be disclosed. Please feel at ease.

We greatly appreciate your time and support for our scientific research!

□ I am aware and agree that the collected data will be used for scientific research.

**Part 1: Basic Information**

1. Your Gender:
   - Male
   - Female
2. Your Age: ______ years old
3. Your Occupation:
   - Doctor
   - Nurse
   - Technician
   - Engineer
   - Other
4. Your Education Level:
   - College and below
   - Undergraduate
   - Postgraduate and above
5. Your Professional Title:
   - Licensed Doctor/Nurse and below
   - Attending Doctor/Nurse Supervisor
   - Chief/Deputy Chief Doctor/Nurse
   - Other (Please specify: ______)
6. Years working in the oncology department:
   - 1-5 years
   - 5-10 years
   - 10-20 years
   - Over 20 years
7. The level of your healthcare institution:
   - Tertiary A
   - Tertiary
   - Secondary A
   - Secondary and below
8. Have you received training related to bone scanning technology and protection?
   - Yes
   - No
9. Do you come into contact with bone scan patients during your routine clinical practice?
   - Yes
   - No

**Part 2: Knowledge of Bone Scans (SPECT)**

1. Are you familiar with the basic principles of bone scans (SPECT)?
   - Yes
   - Not completely
   - A little
   - Not at all
2. Do you know the advantages and limitations of bone scans?
   - Yes
   - No
3. In cancer diagnosis, in which situations do you think bone scans should not be performed?
   - Bone metastasis evaluation
   - Bone pain assessment
   - Bone tumor assessment
   - Treatment effectiveness evaluation
   - Screening of asymptomatic patients
4. Does tumor tissue absorb more tracer?
   - Yes
   - No
5. Which types of cancer are most likely to cause bone metastasis? (Select multiple)
   - Breast cancer
   - Lung cancer
   - Liver cancer
   - Pancreatic cancer
   - Colon cancer
6. To minimize radiation exposure to patients and medical staff, what is the optimal scanning time range?
   - Less than 10 minutes
   - 10-30 minutes
   - 30-60 minutes
   - More than 60 minutes
7. Is it necessary to conduct regular bone scans in both early and late stages of breast cancer?
   - Incorrect
   - Correct
   - Not sure
8. Injecting radionuclides for bone scans poses a significant radiation hazard to the patient’s body?
   - Incorrect
   - Correct
   - Not sure
9. Patients undergoing bone scans carry radioactive material and should avoid close contact with pregnant women and infants?
   - Incorrect
   - Correct
   - Not sure
10. How far should bone scan patients stay away from others after the scan?
    - About two fists apart
    - More than 1 meter apart
    - More than 2 meters apart
    - Not sure
11. After a bone scan, is it necessary for the patient to be isolated?
    - Incorrect
    - Correct
    - Not sure
12. Should patients drink more water and urinate more frequently after a bone scan?
    - Incorrect
    - Correct
    - Not sure
13. The rate of metabolism of the radioactive substance in bone scan patients is approximately:
    - About 1 week
    - About 2 days
    - About half a month
    - Not sure
14. Does renal insufficiency affect the results of bone scans?
    - Yes
    - No
    - Not sure
15. If a patient has taken bismuth-containing medication (such as Pepto-Bismol) or used barium contrast for an X-ray in the past 4 days, can they undergo a bone scan?
    - Yes
    - No
    - Not sure

**Part 3: Attitudes Towards Bone Scans (SPECT)**

1. How do you value the application of bone scans in cancer diagnosis and treatment?
   - Very valuable
   - Valuable
   - Neutral
   - Not valuable
   - Completely worthless
2. Are you willing to recommend or participate in using bone scan technology?
   - Very willing
   - Willing
   - Neutral
   - Unwilling
   - Very unwilling
3. Do you think bone scans help improve diagnostic efficiency and treatment outcomes for cancer patients?
   - Very helpful
   - Somewhat helpful
   - A little helpful
   - Barely helpful
   - Not helpful at all
4. Do you believe the radiation risks of bone scans are worth attention?
   - Yes
   - No
5. After a bone scan, do you worry that the radiation carried by the patient might affect your health?
   - Not worried at all
   - Not worried
   - Neutral
   - Worried
   - Very worried
6. Do you think the number of bone scans can be reduced?
   - Strongly disagree
   - Disagree
   - Neutral
   - Agree
   - Strongly agree
7. Do you believe other examination methods such as CT or MRI are safer and can replace bone scans?
   - Strongly disagree
   - Disagree
   - Neutral
   - Agree
   - Strongly agree
8. Do you feel that the radiation protection measures for routine bone scan procedures are sufficient?
   - Yes
   - No
9. Do you believe bone scan (SPECT) technology will play a more important role in future clinical practice?
   - Yes
   - No

**Part 4: Practice of Bone Scans (SPECT)**

1. Have you used bone scan (SPECT) technology in your clinical practice?
   - Yes
   - No
2. Do you explain the radiation safety and protective measures of bone scans to patients during communication?
   - Always
   - Usually
   - Occasionally
   - Never
3. Do you combine explanations with other imaging examinations?
   - Always
   - Usually
   - Occasionally
   - Never
4. Do you take appropriate precautions when operating bone scans or when aware of a patient undergoing a bone scan?
   - Yes
   - No
5. Do you have strict preparation and procedural guidelines before performing a bone scan (SPECT)?
   - Yes
   - No
6. Have the results of bone scans significantly influenced your clinical diagnostic decisions?
   - Yes
   - No
7. Have you received training in accident and emergency response related to bone scans (SPECT)?
   - Yes
   - No
